# Supplementary material for: Characterizing common loss-of-function genes and their potential utility in assessing population variability and chemical susceptibility
Source: bioRxiv. 2025 Dec 19:2025.12.16.694775. Preprint. [Version 1] doi: 10.64898/2025.12.16.694775 (PMC12746020; doi:10.64898/2025.12.16.694775)
Supplement: Supplement 1 [file media-1.pdf]

Supplementary Figures.

Title: Characterizing common loss-of-function genes and their potential utility in assessing population variability and chemical susceptibility

Chanhee Kim[1], Zhaohan Zhu[2], Abderrahmane Tagmount[1], W. Brad Barbazuk[3,4], Rhonda Bacher[2], Leah D Stuchal[1], Christopher Martyniuk[1], and Christopher D. Vulpe[1*]

[1]Center for Human and Environmental Toxicology, Department of Physiological Sciences, College of Veterinary Medicine, University of Florida, Gainesville, FL, United States

[2]Department of Biostatistics, University of Florida, FL, United States

[3]Department of Biology, University of Florida, FL, United States

[4]University of Florida Genetics Institute, University of Florida, FL, United States

*Correspondence: Christopher D Vulpe, M.D., Ph.D. (e-mail: cvulpe@ufl.edu); Center for Human and Environmental Toxicology, Department of Physiological Sciences, College of Veterinary Medicine, University of Florida, Gainesville, Florida, United States

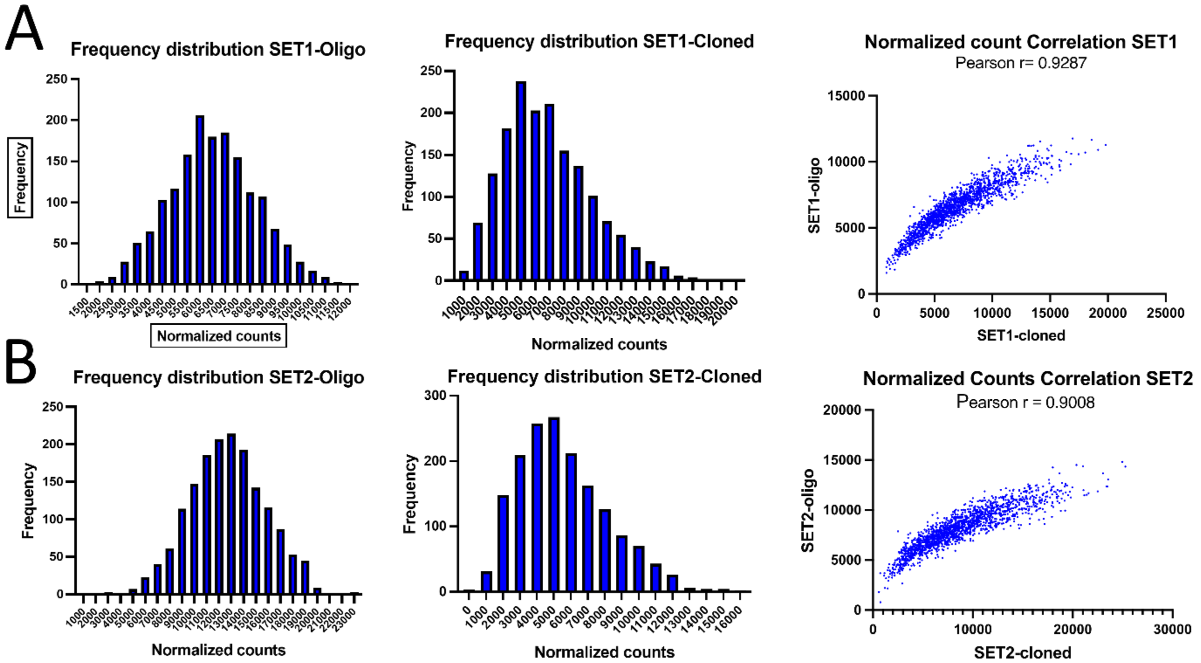

Figure S1. Quality assessment of the custom sgRNA libraries (Set1 and Set2) was conducted. The distribution of normalized sgRNA counts and their corresponding frequencies in the library before and after

cloning (Oligo-pool vs. cloned) are shown in panels A and B. The correlation between the Oligo-pool and the cloned library is presented with the Pearson correlation coefficient (r) in panel C.

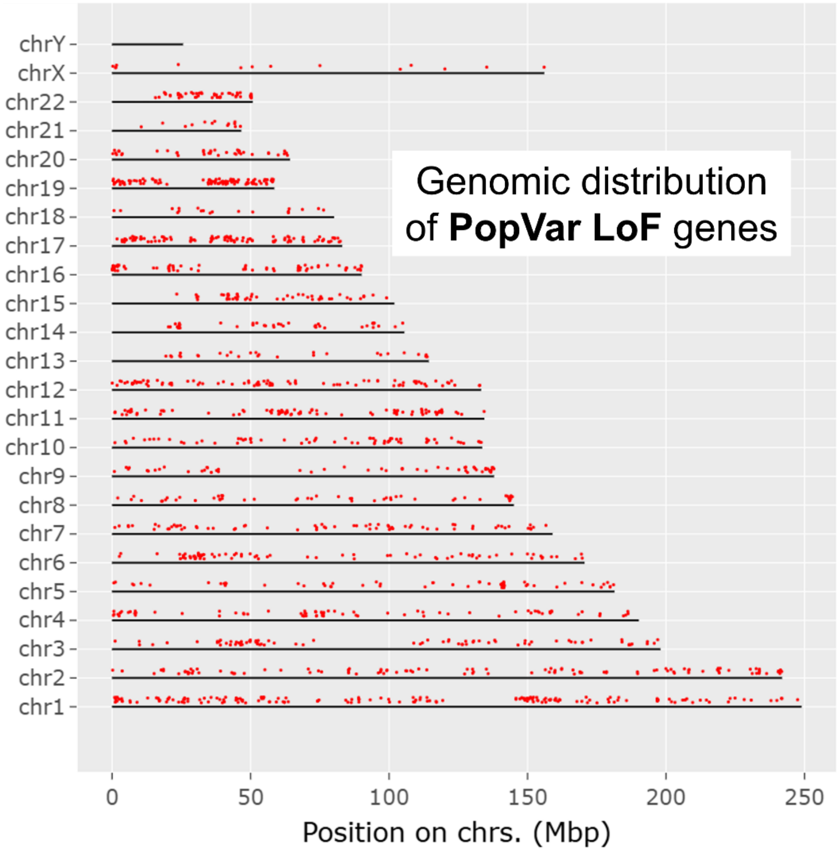

Figure S2. Distribution of individual PopVarLoF genes across chromosomes.

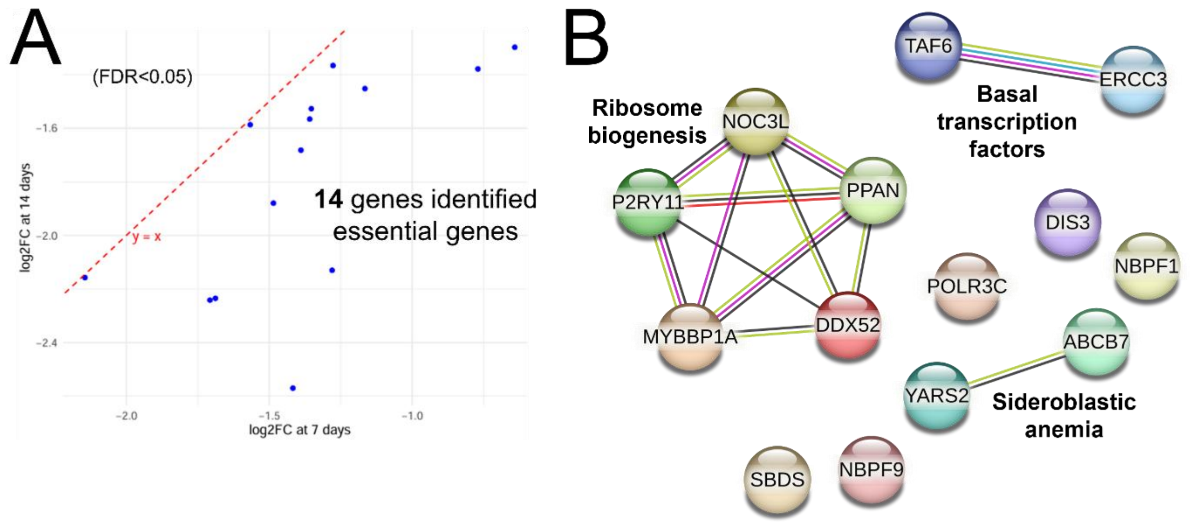

Figure S3. Empirically identified essential genes (14 genes) in the PopVar LoF CRISPR screening in a time-course manner (7 doublings: T_7 and 14 doublings: T_{14}). (A) Blue dots indicate 14 essential PopVarLoF genes which showed increased depletion between T_7 and T_{14}. (B) STRING functional enrichment of the 14 essential genes. Three clusters were identified as presented Ribosome biogenesis, Basal transcription factors, and Sideroblastic anemia. Detailed features on these 14 genes are available in Table S3.
